# Reconstructing feedback representations in the ventral visual pathway with a generative adversarial autoencoder

**DOI:** 10.1371/journal.pcbi.1008775

**Published:** 2021-03-24

**Authors:** Haider Al-Tahan, Yalda Mohsenzadeh

**Affiliations:** 1 Department of Computer Science, The University of Western Ontario, London, Ontario, Canada; 2 Brain and Mind Institute, The University of Western Ontario, London, Ontario, Canada; Oxford University, UNITED KINGDOM

## Abstract

While vision evokes a dense network of feedforward and feedback neural processes in the brain, visual processes are primarily modeled with feedforward hierarchical neural networks, leaving the computational role of feedback processes poorly understood. Here, we developed a generative autoencoder neural network model and adversarially trained it on a categorically diverse data set of images. We hypothesized that the feedback processes in the ventral visual pathway can be represented by reconstruction of the visual information performed by the generative model. We compared representational similarity of the activity patterns in the proposed model with temporal (magnetoencephalography) and spatial (functional magnetic resonance imaging) visual brain responses. The proposed generative model identified two segregated neural dynamics in the visual brain. A temporal hierarchy of processes transforming low level visual information into high level semantics in the feedforward sweep, and a temporally later dynamics of inverse processes reconstructing low level visual information from a high level latent representation in the feedback sweep. Our results append to previous studies on neural feedback processes by presenting a new insight into the algorithmic function and the information carried by the feedback processes in the ventral visual pathway.

## Author summary

It has been shown that the ventral visual cortex consists of a dense network of regions with feedforward and feedback connections. The feedforward path processes visual inputs along a hierarchy of cortical areas that starts in early visual cortex (an area tuned to low level features e.g. edges/corners) and ends in inferior temporal cortex (an area that responds to higher level categorical contents e.g. faces/objects). Alternatively, the feedback connections modulate neuronal responses in this hierarchy by broadcasting information from higher to lower areas. In recent years, deep neural network models which are trained on object recognition tasks achieved human-level performance and showed similar activation patterns to the visual brain. In this work, we developed a generative neural network model that consists of encoding and decoding sub-networks. By comparing this computational model with the human brain temporal (magnetoencephalography) and spatial (functional magnetic resonance imaging) response patterns, we found that the encoder processes resemble the brain feedforward processing dynamics and the decoder shares similarity with the brain feedback processing dynamics. These results provide an algorithmic insight into the spatiotemporal dynamics of feedforward and feedback processes in biological vision.

## Introduction

In just a couple of hundred milliseconds, our brain interprets the visual scene around us [1–5], identifies faces [6, 7], recognizes objects [8–13], and localizes them [14–18]. Decades of cognitive neuroscience research have demonstrated that the brain accomplishes these complicated tasks through a cascade of hierarchical processes in the ventral visual stream starting in the early visual cortex (EVC) and culminating in the inferior temporal (IT) cortex.

While the feedforward recruitment of this hierarchy explains the core neural response patterns underlying visual recognition [19–21], it is unable to account for behavioural and neural dynamics observed in years of psychophysical, neurophysiological, and magneto/electrophysiological experiments [12, 22–25]. Indeed, variable timing of neural responses to visual stimuli beyond 200ms has been frequently associated with accumulation of sensory evidence through recurrent processes in the visual brain. However, the precise computational role of neural recurrent/feedback processes remains poorly understood at the system level. In particular, the algorithmic function of feedback processes and the type of information sent back along the visual hierarchy is still unknown.

To address this question, we develop a generative model which is adversarially trained on a diverse set of image categories. The model consists of two sub-networks: (i) An encoder sub-network receives a given visual stimulus, processes it in a hierarchy of neural layers to eventually produce a latent representation (code) of the visual input and (ii) a decoder sub-network which receives the latent representation and aims to reproduce the visual input from the information encoded in the latent representation.

This generative model enables us to not only investigate the encoding process of visual representations along the hierarchy of the encoder sub-network layers but also provides us an insight into the reverse process, i.e., reconstructing the representations along the decoder sub-network layers. We hypothesize that the visual information in the encoder sub-network in our computational model mimics the feedforward pathway in the ventral visual stream and the decoder sub-network which performs the reverse function may reveal the representations along the feedback pathway. To test this hypothesis, after training the proposed model, we compare the representations along its layers with magnetoencephalography (MEG) and functional magnetic resonance imaging (fMRI) data acquired from fifteen human participants in a visual recognition experiment [13].

Our model identified two separate dynamics of representational similarities with MEG temporal data. The first one is consistent with the temporal hierarchy of processes transforming low level visual information into high level semantics in the feedforward sweep, and the second one reveals a temporally subsequent dynamics of inverse processes reconstructing low level visual information from a high level latent representation in the feedback sweep. Further, comparison of encoder and decoder representations with two fMRI regions of interests, namely EVC and IT, revealed a growing categorical representation along the encoder layer (feedforward sweep) similar to IT and a progression in detail visual representations along the decoder layers (feedback sweep) akin to EVC.

## Results

### Construction of a generative model performing image reconstruction

**Fig 1:**
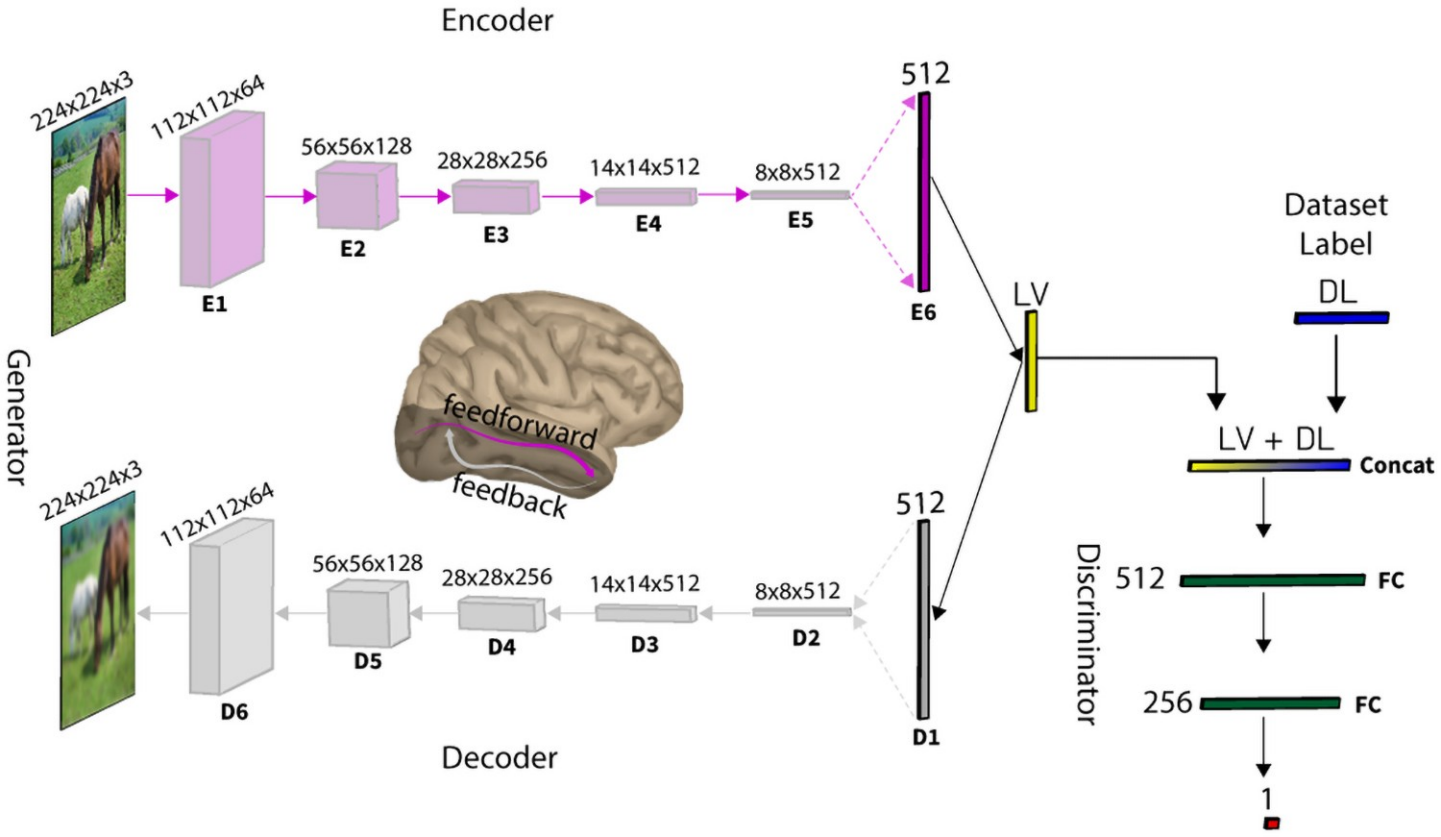
Computational model architecture. The model is a generative adverserial network. The generator is an autoencoder consisting of five convolutional blocks (E1-E5) and one fully connected layer (E6) in the encoder and one fully connected layer (D1) followed by five deconvolutional blocks in the decoder (D2-D6). Each convolutional block encompasses batch normalization, convolution, nonlinear activation function (Leaky Rectified Linear Unit), and pooling operations. Alternatively, each deconvolutional block encompasses batch normalization, transposed convolution, nonlinear activation function (Leaky Rectified Linear Unit), and upsampling operations. The discriminator consists of two fully connected layers. The training Data set consists of 1,980,000 images organized into four super-ordinate categories: (i) Faces, (ii) Animates, (iii) Objects, (iv) Scenes. **LV** denotes the latent vector generated by the encoder and **DL** is a one-hot data set label (one of the four mentioned training data sets). Both vectors are concatenated and fed to the discriminator, while only the latent vector is fed to the decoder.

Previous work revealed that deep convolutional neural networks (DNNs) trained on image classification develop hierarchical representations similar to the cascade of processes along ventral visual pathway [3, 26–33]. However, neuroscience evidence suggests top-down modulations of neural responses which occur after some delay through abundant number of feedback connections in visual cortex are critical to resolving visual recognition in the brain [12, 23, 24]. Therefore, these feedforward deep neural network models do not fully represent the complex visual processes in the ventral visual pathway. Here, we investigate whether a deep generative model trained to compress and reconstruct images could reveal similar representations as feedforward and feedback processes in the ventral visual pathway. With this aim, we developed a deep generative autoencoder neural network model using adverserial autoencoder (AAE) framework [34]. AAE is a generative adverserial network (GAN) [35] where the generator has an autoencoder architecture. Fig 1 depicts our proposed model architecture. The autoencoder generator consists of two main components: 1) an encoder which receives the visual stimuli and performs a cascade of simple operations such as convolution, pooling, and normalization to map the visual input to a latent feature vector (LV); 2) a decoder which receives the latent vector and performs a cascade of simple transposed convolution operations to reconstruct the input visual stimuli from information encoded in the latent space. The model is trained with two objectives—a reconstruction loss criterion, and an adversarial criterion. The dual objectives training turns the autoencoder into a generative model whose latent space learns data distribution properties that enables generative process and avoids overfitting to the reconstruction objective. We hypothesize that the encoder sub-network models the feedforward pathway of processes in ventral visual stream, while the decoder sub-network models the reconstruction of visual features in the feedback pathway.

**Fig 2:**
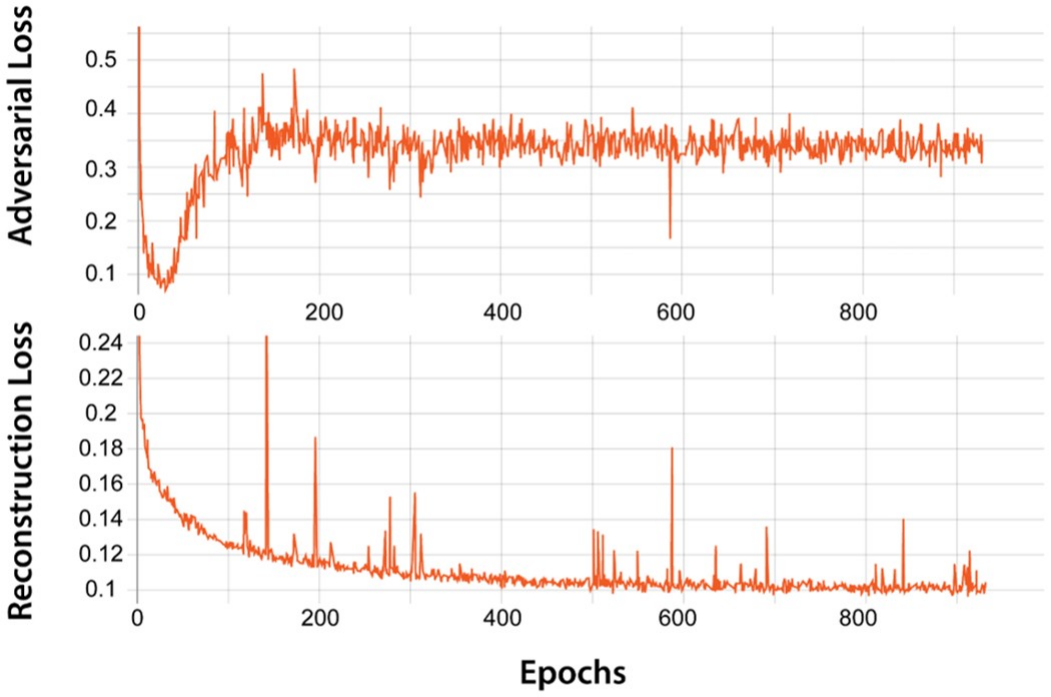
Computational model performance. (A) Adversarial and Reconstruction loss over training epochs.

To train our model, we assembled a super category data set (see Materials and methods section for details). The super category data set includes 1,980,000 images from four equally distributed categories of (1) Faces, (2) Animates, (3) Objects, and (4) Scenes. The rational behind assembling and using this data set is two-fold: (1) ecologically, the human brain learns to develop high-level category representations across multiple recognition tasks (e.g. faces, animals, objects, scenes, etc.); Indeed, years of neuroscience research have identified a cascade of brain regions along ventral visual stream starting in early visual cortex (EVC) which processes low level visual features and eventually resolves visual categories at the end of the hierarchy in inferior temporal cortex (IT). (2) In this study, we compared model representations with brain imaging data (fMRI and MEG) from a visual recognition experiment [13]. We maintained consistency with the four categories from the stimulus set utilized in the brain imaging experiment which includes 156 images. Please note that this stimulus set was not used for training the model. After training the model on super category data set for over 800 epochs when the adverserial and reconstruction losses reached their local minima on the training set (Fig 2A), we determined how well our model performed on the 156 image set (as a testing dataset). The model performed the reconstruction on the training dataset with 0.109 ± 0.006 mean absolute error (MAE) and on the 156 image set with 0.119 ± 0.003 MAE between the input image and the corresponding reconstructed image. We computed the upper-bound performance of the model by sampling random pairs of images from the training and testing sets performing MAE between the different image pairs. On the training set, we observed a MAE of 0.53 ± 0.014 between the random image pairs and 0.62 ± 0.017 on the random pairs from the testing set. Furthermore, we computed the MAE loss on training and testing sets with the same model architecture but with random weights. On the training and testing sets, we obtained a MAE of 0.49 ± 0.024 and 0.46 ± 0.009, respectively. These results show that the model not only have converged to an optimum but also generalizes well to the testing set.

### Representational similarity of the generative model to early and late brain regions in the ventral visual stream

We first determined the encoder/decoder representational similarities with early and late brain regions along the ventral visual cortex. For this, we chose early visual cortex (EVC) and inferior temporal cortex (IT) defined anatomically based on [36] and employed the representational similarity analysis (RSA) method [37, 38] as the integrative framework for model-brain comparisons.

**Fig 3:**
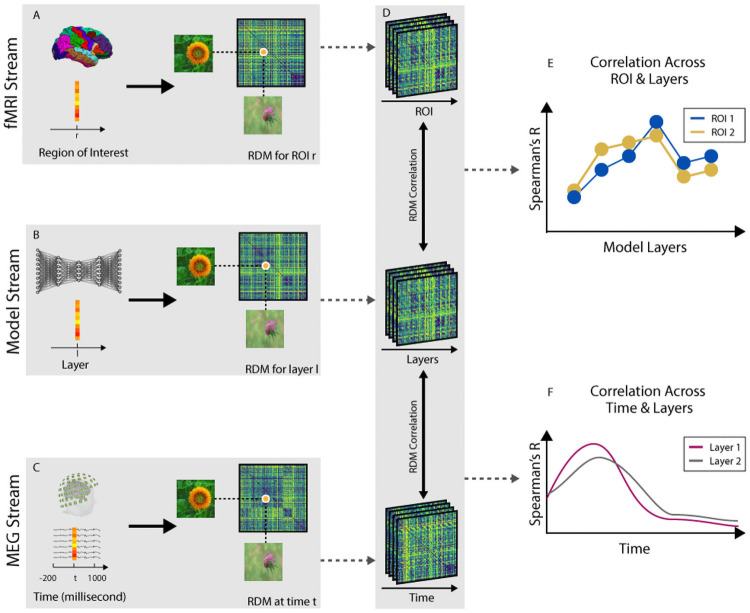
Representational similarity analysis to compare fMRI, MEG and model representations. (A) fMRI response patterns were extracted from each ROI and pairwise condition-specific dissimilarities (1-Pearson’s R) were computed to create one fMRI RDM per ROI and participant (see Materials and methods section for detail). (B) RDMs for the generative model were computed at each convolutional/deconvolutional block after feeding 156 images to the computational model. (C) MEG data consists of time-series data with 306 channels and 1200 time points (milliseconds) per trial. For each condition, we extracted a vector of size 306 at each time point as the whole brain activity pattern to compute the RDMs using SVM classifiers decoding accuracies (see Materials and methods section for detail). (D) Using RDMs from MEG and fMRI ROIs, we compared (Spearman’s R) them with the RDMs from the computational model to investigate the spatio-temporal correspondences between the human brain and the computational model. (E) Correlations between ROI fMRI RDMs and computational model RDMs result in a subject-specific correlation value for each ROI across model layers, which we then average them over subjects. (F) Correlations between time-resolved MEG RDMs and computational model RDMs result in a subject-specific signal for each layer across time, which we then average them over subjects.

**Fig 4:**
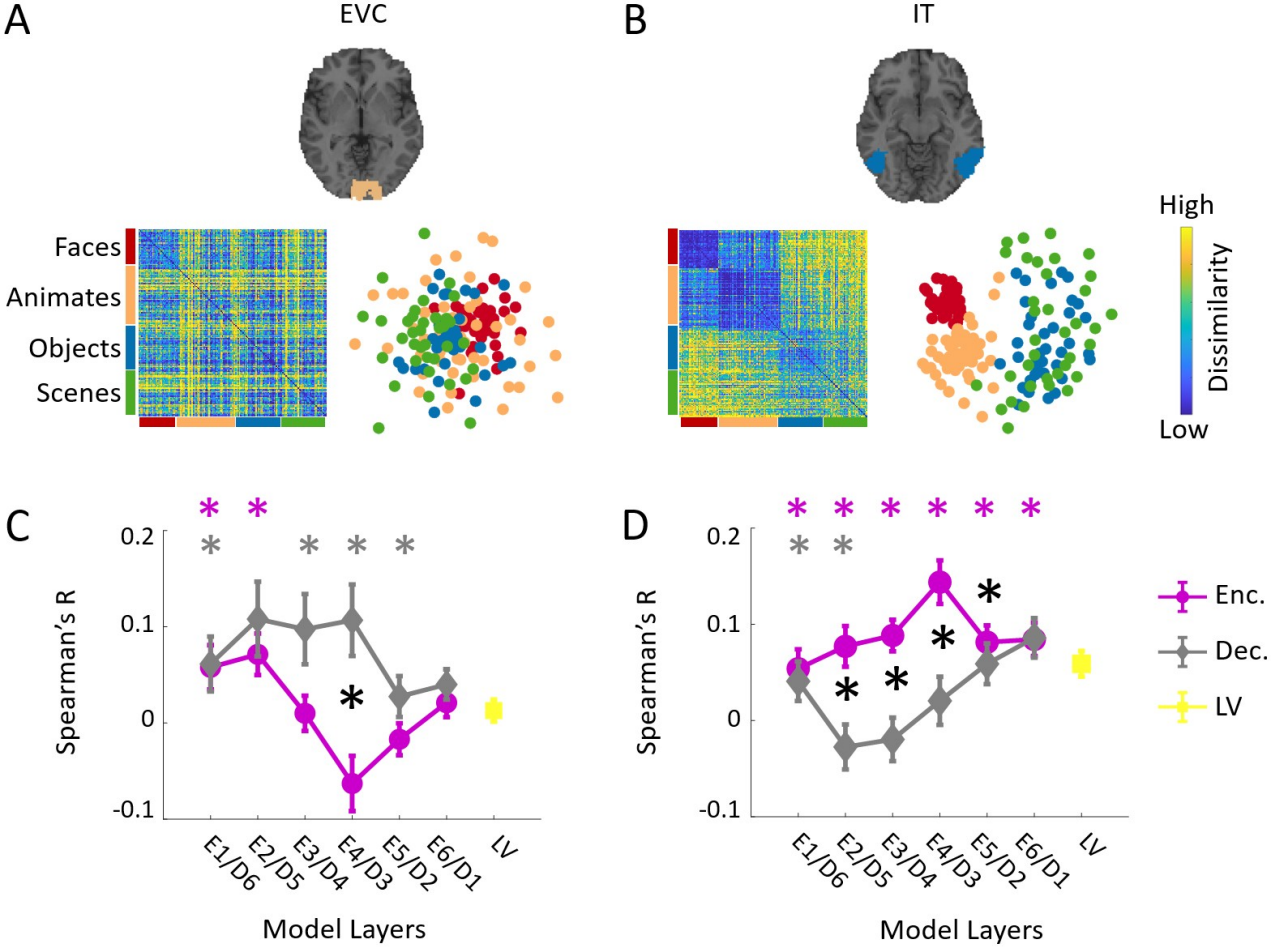
Spatial representational comparisons. (A) Neural representations in early visual cortex (EVC). The subject-averaged EVC RDM matrix, and its 2D multidimensional scaling visualization. (B) Neural representations in inferior temporal area (IT). The subject-averaged IT RDM matrix, and its 2D multidimensional scaling visualization. (C) Encoder, decoder and LV layer RDMs are correlated (Spearman’s R) with subject-specific EVC RDMs. The averaged correlations over subjects with standard error of the mean are depicted. (D) Encoder, decoder and LV layer RDMs are correlated (Spearman’s R) with subject-specific IT RDMs. The averaged correlations over subjects with standard error of the mean are depicted. The color coded (*) above each panel in C-D indicates that the correlation of the corresponding layer is significantly above zero. The black (*) indicates the correlations of the corresponding encoder and decoder layers are significantly different (N = 15; two-sided ttests; false discovery rate corrected at *P* < 0.05).

For each region of interest (ROI), we extracted the fMRI response patterns to each image, vectorized it and computed condition-specific pairwise distances (1- Pearson’s R) to create a 156 x 156 representational dissimilarity matrix (RDM) per participant. We also fed the images to the generator and extracted layer-specific activations for each image condition. Then by computing the pairwise distances (1-Pearson’s R) of image evoked layer activation patterns, we created layer-specific RDMs (see Fig 3 and Materials and methods section for details). We then compared subject-specific ROI RDMs with the model layer RDMs by computing Spearman’s correlations (Fig 3A, 3D and 3E). Fig 4A and 4B show subject averaged RDMs and their corresponding 2-dimensional multidimensional scaling (MDS) visualizations of EVC and IT, respectively. As expected EVC shows a random pattern across categories, whereas IT demonstrates clear categorical distinctions. Fig 4C and 4D compares the encoder and decoder layers correlations with EVC and IT RDMs. The EVC representational correlations across layers of the encoder (and inverse order of the decoders) demonstrate a decreasing trend, while IT representational correlations across model layers progressively increase. Notably, early processing layers of the model (E1/D6) and late processing layers (E6/D1) show similar correlations both for EVC and IT. However, middle layers correlations are significantly different when comparing encoder and decoder in both ROIs (N = 15; two-sided ttests; false discovery rate corrected at *P* < 0.05). Further, the correlations of EVC is stronger with the decoder than encoder whereas the correlations of IT is higher for the encoder than the decoder. This indicates the reconstructing processes are more similar to detail representations in EVC than IT.

### The generative model unfolds the temporal dynamics of brain feedforward and feedback representations

**Fig 5:**
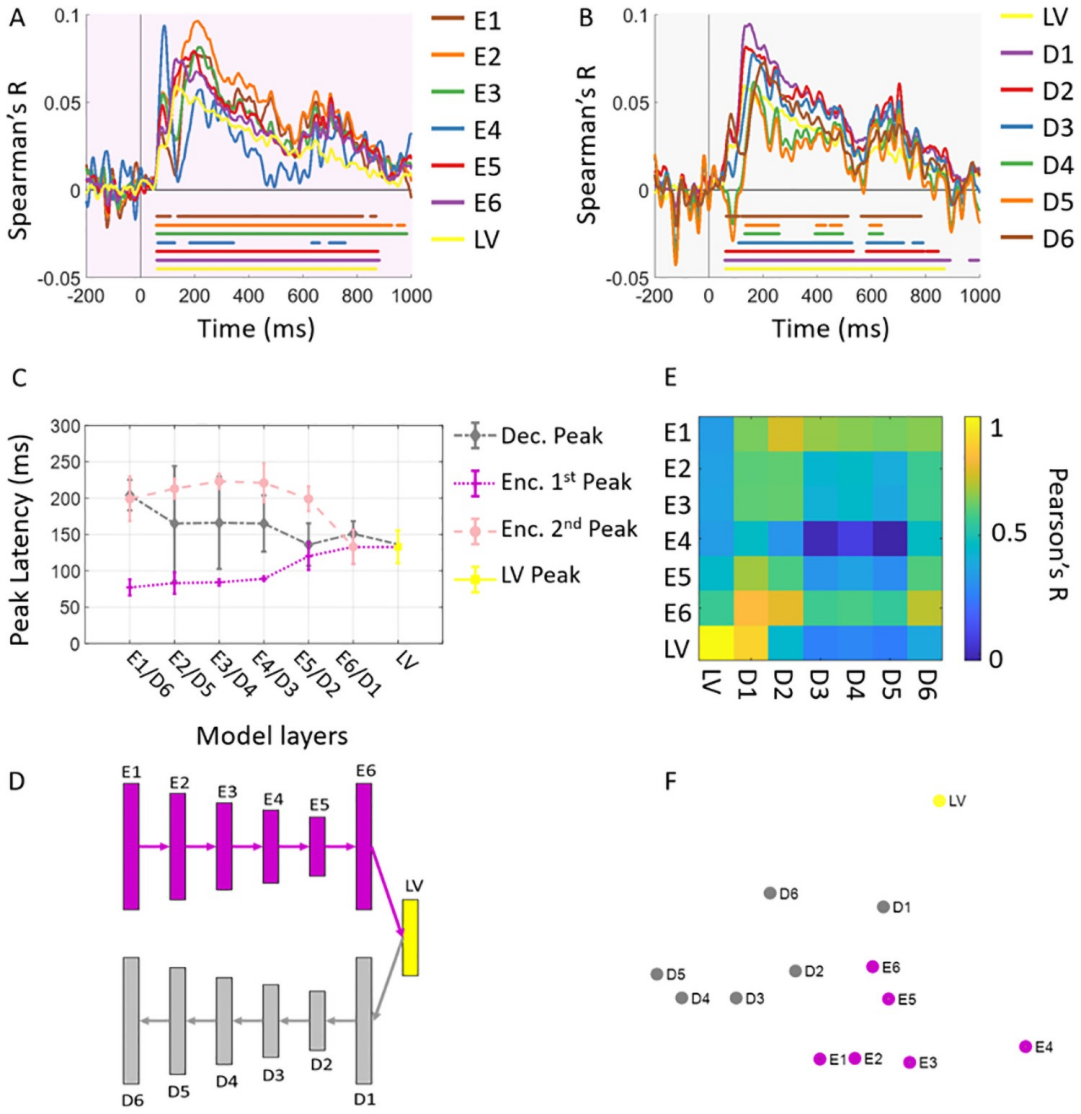
Temporal representational comparisons. (A) Encoder and MEG representational comparison. We correlated the encoder layer RDMs with subject-specific time-resolved MEG RDMs resulting in fifteen correlation time courses. We then averaged these time courses over participants. (B) Decoder and MEG representational comparison. Correlation of the decoder layer RDMs and time-resolved MEG RDMs. The color-coded lines below the curves show the time points when the correlations are significantly above zero (N = 15; permutation tests; cluster definition threshold *P* < 0.01; cluster threshold *P* < 0.05). (C) Peak latency for encoder and decoder. The encoder have significantly earlier peak latency across all layers (*P* = 0.014). Error bars are expressed in standard error of the mean. (D) The architecture of the models with layers’s label corresponding to (C). (E) The visualization of relationships between model layers representations. The matrix of RDM correlations between encoder and decoder layers is depicted. Each matrix entry compares two RDMs indexed by corresponding row and column in terms of Pearson’s R. (F) The multidimensional scaling visualization of the RDMs relationships.

The visual information traverses a hierarchy of regions in the visual cortex and evolves over time rapidly. While the structure of our proposed model does not directly delineate the human brain temporally, it has a clear sequential structure which temporally unfolds the feedforward and feedback sequences in the visual cortex. That is, information flows layer to layer and evolves from image low level features to higher level latent concepts (feedforward sweep) and then from this high level latent code sequentially the image level information is reconstructed (feedback sweep). To test the hypothesis that the proposed model indeed temporally mirrors ventral visual stream dynamics, we compared the representations of time-resolved MEG data acquired in a visual recognition experiment with the layer representations in the encoder and decoder sub-networks. From each participant’s data, we first extracted the MEG sensor measurements for each image condition from -200ms to 1000ms (with 1 ms resolution) relative to image onset. Then we computed dissimilarities (SVM classifiers decoding performances, see Materials and methods section for details) between evoked MEG pattern vectors of each pair of images and created time resolved representational dissimilarity matrices (RDMs) for each individual. We also fed the images to the generator and extracted layer-specific activations for each image condition. Then by computing the pairwise distances (1-Pearson’s R) of image evoked layer activation patterns, we created layer-specific RDMs (see Fig 3 and Materials and methods section for details). Next we correlated layer-specific model RDMs with time-resolved subject specific MEG RDMs (Spearman’s R) resulting in correlation time series for each layer of the model. Fig 5A and 5B show the subject averaged correlation time series for layers of encoder and decoder sub-networks, respectively. Our results showed that all layers of the proposed model were representationally similar to human brain activity patterns, indicating that the model captures evolution of brain visual representations over time (N = 15; permutation tests; cluster definition threshold *P* < 0.05; cluster significance threshold *P* < 0.01). Next, we investigated whether the hierarchy of the layered architecture unfolds the temporal dynamics of encoding (feedforward) and decoding (feedback) visual processes in the brain. Specifically, we examined the relationship between hierarchy of model layers and the peak latency of the correlation time series (Fig 5C). Consistent with previous works [29], the first peak latency of correlation time courses relating MEG and the encoder representations increased with the hierarchy of the encoder layers (Spearman’s *R* = 0.78, *P* < < 0.0001). The inspection of peak latencies in the decoder time series depicted in Fig 5B revealed a progressively increasing pattern from D1 to D6 (Spearman’s *R* = 0.84, *P* < < 0.0001). That is, the peak latency in D1 occurs at 151ms and over the decoder layers progressively the peak latency increases, the peak latency of D6 occurs at 204ms. Given that the decoder later layers are reconstructing the visual stimuli and thus closer to image level feature space, these temporal dynamics may explain the temporal dynamics of feedback information sent down the ventral stream. We further identified a salient second peak in layers E1 to E5 which their peak latencies negatively correlated with layers order in the encoder (Spearman’s *R* = −0.57, *P* < < 0.0001) and they are significantly later than the corresponding layers in the decoder (all peak latency analyses are based on permutation based bootstrapping; N = 15; two-sided hypothesis tests; *P* < < 0.0001; Bonnferoni corrected). Again this confirms later dynamics of representational similarities between these layers and the brain, possibly indicating a second sweep of visual information processing.

In the next step, we investigated the representational relationships among the encoder and the decoder layers of the model. To this end, we first computed the pairwise correlations between all encoder and decoder layer RDMs. The matrix depicted in Fig 5E summarizes these pairwise correlations and reveals which representations across model layers are similar or dissimilar. Fig 5F visualizes these relationships with multi dimensional scaling (MDS) method. Visual inspection of this matrix manifests firstly the dissimilarity of latent layer representation from encoder and decoder layers, secondly similarity of late layer of the encoder (E6) and early layer of the decoder (D1); and also similarity of early layer of decoder (E1) and late layer of the decoder (D6), thirdly the dissimilarity of middle layers of encoder and decoder indicating the difference in the representations of encoding (feedforward) and decoding (feedback) processes.

**Fig 6:**
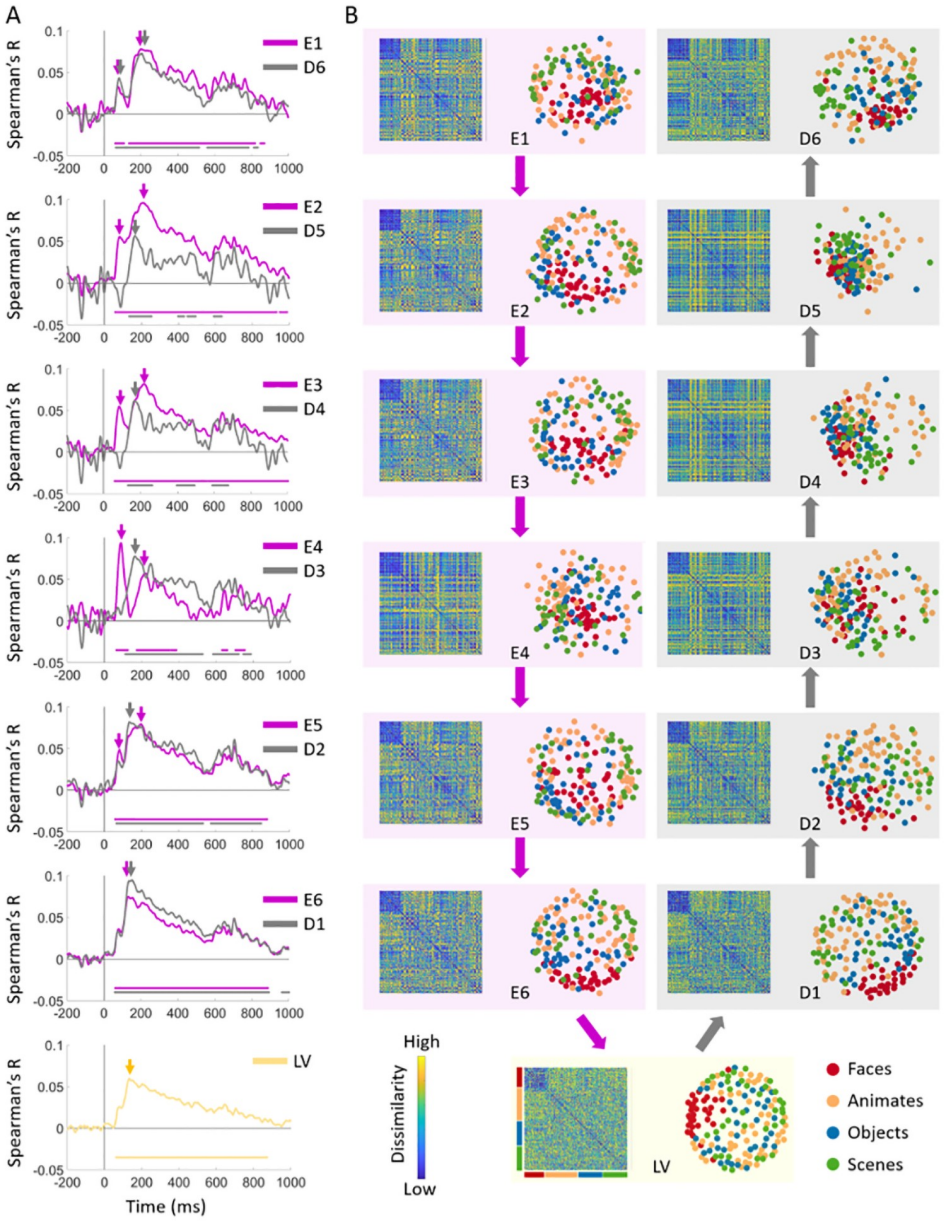
Comparisons of encoder and decoder representational dynamics. (A) Comparison of correlation time series of the encoder and decoder layers with the same level of processing. The color-coded lines below the curves show the timepoints when the correlations are significantly above zero (N = 15; permutation tests; cluster definition threshold *P* < 0.01; cluster threshold *P* < 0.05). (B) The model RDMs and their corresponding MDS visualizations.

To obtain a more clear picture of brain-model temporal dynamics relationships across encoder and decoder sub-networks, we compared the correlation time courses corresponding to the encoder and decoder layers with the same level of processing in Fig 6A. The correlation time course of latent layer is depicted separately. Fig 6B depicts the corresponding model layers RDMs and their 2-dimensional MDS visualizations. As demonstrated in Fig 6, firstly the low level feature processing layer of encoder (E1) and decoder (D6) follow a notably similar dynamics. Further, the high level feature processing layers of encoder (E6 and E5) also depict a similar temporal dynamics with high level feature processing layers of the decoder (D1 and D2, respectively). However, the dynamics are explicitly different when middle level layers are compared (i.e. E2 vs.D5, E3 vs. D4, E4 vs. D3). This is also evident from comparison of the MDS visualizations of RDMs in each row. The peak latencies of the correlation time courses are marked with color-coded arrows. Consistent with Fig 5C, the peak latencies of low and high level processing layers coincide around the same time, whereas the peaks of mid-level feature processing layers of the decoder occurs between the first and second peaks of the corresponding encoder time courses.

Together, comparison of MEG temporal representations with the encoder and decoder sub-networks of our proposed model segregated the brain representational dynamics that transforms the low level visual features to high level categorical semantics and the inverse functional processes that reconstructs low level features from the high level code. These two identified dynamics of processing can be associated with feedforward and feedback sweeps along ventral visual stream.

### Factors determining the similarity between the generative model and spatiotemporal neural dynamics of visual process

The significant correlation of the proposed model layers and temporal and spatial brain data with the dynamics described in previous sections raises the critical question of the origin of this relationship. Architecture and training procedure are two fundamental factors that shape any neural network model characteristics. To understand the emergence of this relationship between the brain and our proposed model, we created two alternative models and trained them on the same training image dataset (super category dataset). The first alternative model is a regular autoencoder neural network with the same architecture as the autoencoder in our generative model. In other words, we ablated the discriminator of our model, and trained the autoencoder using only the reconstruction loss. We reasoned that this alternative model would reveal the effect of training procedure, especially the generative property of the model and training with adversarial loss. The second alternative model that we investigated has the same architecture as our proposed model but it is untrained (i.e., random connection weights). This model would reveal the effect of the model architecture alone without training.

**Fig 7:**
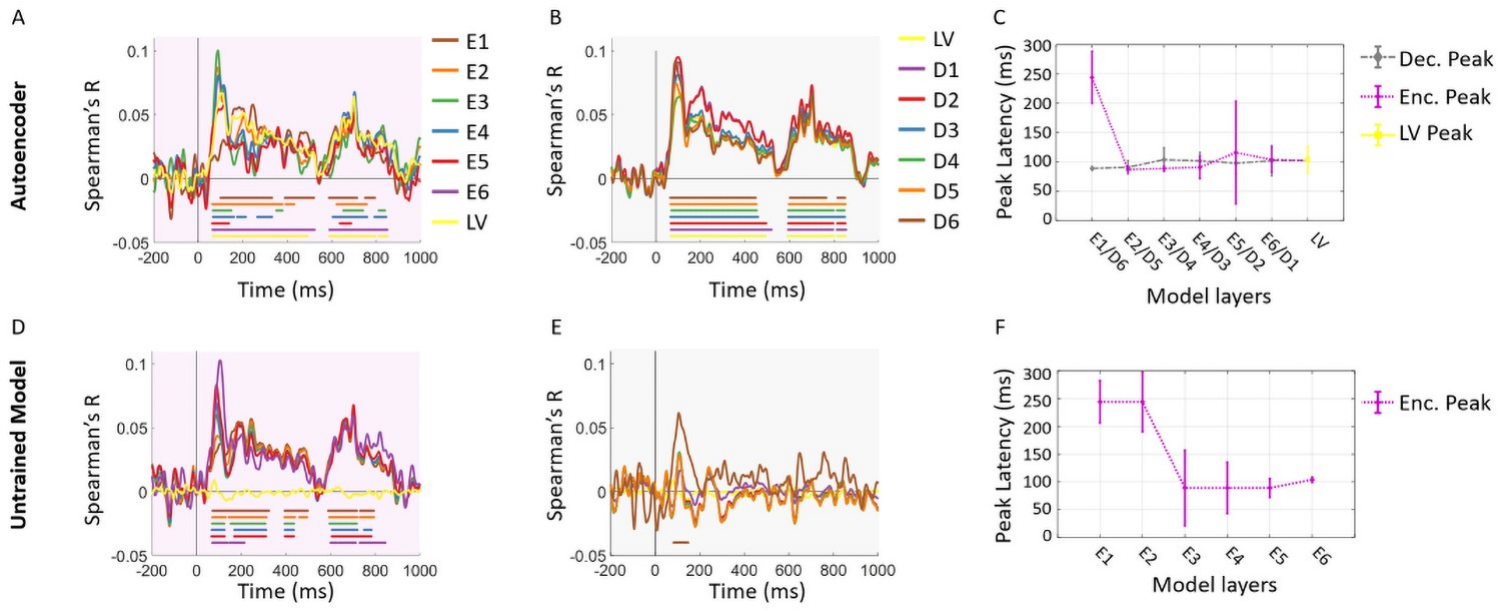
The impact of architecture and training procedure on the representational similarity of the model and brain temporal data. (A) Comparison of the encoder layers of the autoencoder model with MEG representations. We correlated the encoder layer RDMs with subject-specific time-resolved MEG RDMs resulting in fifteen correlation time courses. We then averaged these time courses over participants. (B) Comparison of the decoder layers of the autoencoder model with MEG representations. Correlation of the decoder layer RDMs and time-resolved MEG RDMs. The color-coded lines below the curves show the time points when the correlations are significantly above zero (N = 15; permutation tests; cluster definition threshold *P* < 0.01; cluster threshold *P* < 0.05). (C) Peak latency for encoder and decoder of the autoencoder model. Error bars are expressed in standard error of the mean. (D) Comparison of the encoder layers of the untrained model with MEG representations. We correlated the encoder layer RDMs with subject-specific time-resolved MEG RDMs resulting in fifteen correlation time courses. We then averaged these time courses over participants. (E) Comparison of the decoder layers of the untrained model with MEG representations. Correlation of the decoder layer RDMs and time-resolved MEG RDMs. The color-coded lines below the curves show the time points when the correlations are significantly above zero (N = 15; permutation tests; cluster definition threshold *P* < 0.01; cluster threshold *P* < 0.05). (F) Peak latency for encoder and decoder of the untrained model. Error bars are expressed in standard error of the mean.

**Fig 8:**
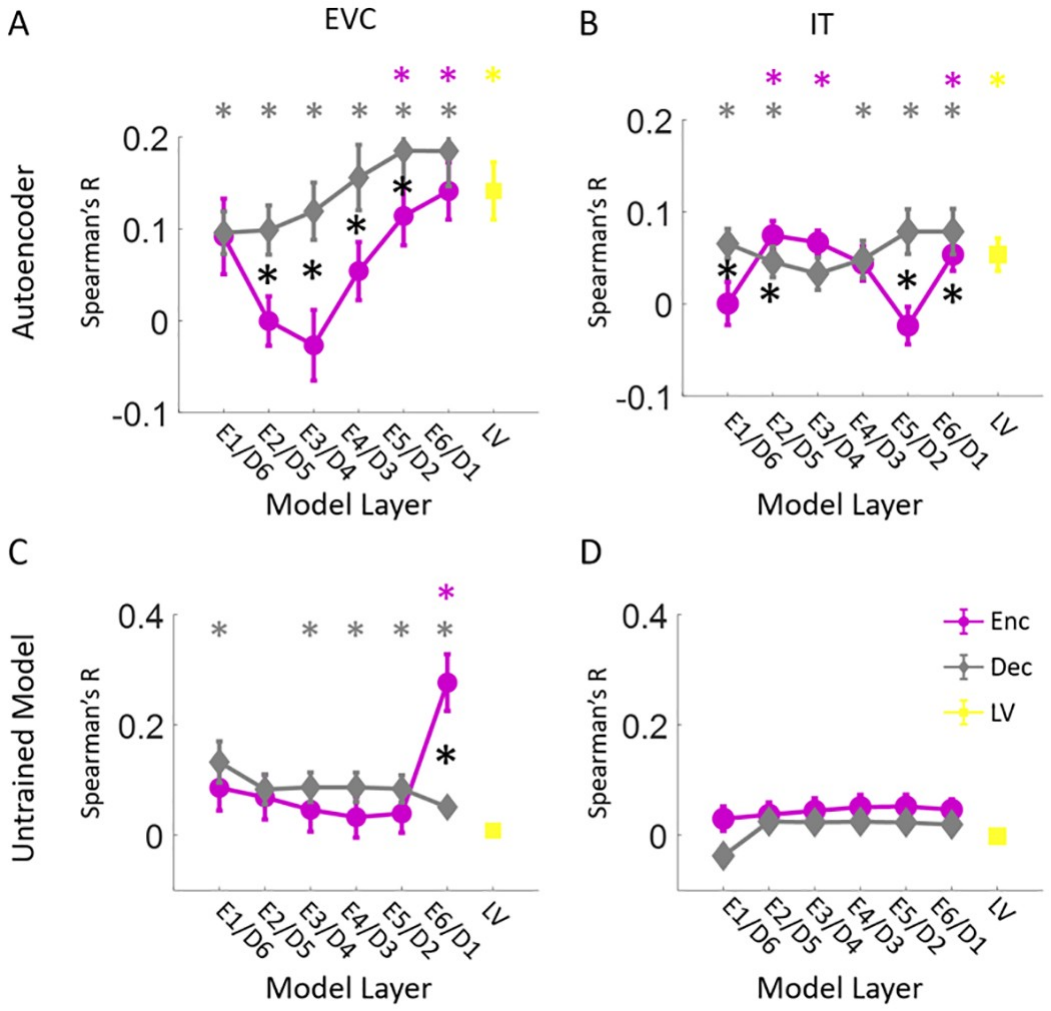
The impact of architecture and training procedure on the representational similarity of the model and brain spatial data. (A) Encoder, decoder and LV layer RDMs of the autoencoder model are correlated (Spearman’s R) with subject-specific EVC RDMs. The averaged correlations over subjects with standard error of the mean are depicted. (B) Encoder, decoder and LV layer RDMs of the autoencoder model are correlated (Spearman’s R) with subject-specific IT RDMs. The averaged correlations over subjects with standard error of the mean are depicted. (C) Encoder, decoder and LV layer RDMs of the untrained model are correlated (Spearman’s R) with subject-specific EVC RDMs. The averaged correlations over subjects with standard error of the mean are depicted. (D) Encoder, decoder and LV layer RDMs of the untrained model are correlated (Spearman’s R) with subject-specific IT RDMs. The averaged correlations over subjects with standard error of the mean are depicted. The color coded (*) above each panel in C-D indicates that the correlation of the corresponding layer is significantly above zero. The black (*) indicates the correlations of the corresponding encoder and decoder layers are significantly different (N = 15; two-sided ttests; false discovery rate corrected at *P* < 0.05).

To assess the spatial and temporal relationships between the two alternative models and the human brain, we fed the 156 image set to these models, extracted image-specific activation patterns from each layer, and computed layer-specific RDMs for each model. We then compared these RDMs with brain MEG RDMs (Fig 7) and fMRI ROI RDMs (Fig 8) by computing their corresponding Spearman’s correlations.

Fig 7A, 7B and 7C show the temporal relations of MEG data with the autoencoder model. As depicted in this figure, the encoder, latent vector, and decoder representations correlated significantly with the time-resolved MEG representations. However, the peak latencies in Fig 7C showed a reverse hierarchy along the encoder layers (*R* = −0.53, *P* = 0.27), and approximately simultaneous peaks in the decoder layers. These finding demonstrate while the ablated model (i.e. when the discriminator is removed) still shows similarity to the temporal visual representations in the brain, the adversarial training is crucial in emergence of the specific temporal dynamics that disentangles feedforward and feedback brain representations in the model.

We also compared the EVC and IT representations with this autoencoder model. We found significant correlations between the encoder layers (E5-E6) and EVC, and between the decoder layers (D1-D6) and EVC (Fig 8A). But the hierarchical relations between EVC and the decoder were reversed (*R* = −0.96, *P* = 0.001) compared to our proposed generative model (Fig 4C). The encoder/decoder layers showed significant correlations with IT representations (Fig 8B) but without hierarchical relations. This shows that the adversarial training is crucial for the emergence of hierarchical correspondences between the model and the brain representations.

Fig 7D, 7E and 7F show the temporal correlations of MEG data with the untrained model. The encoder subnetwork (E1-E6) correlated significantly with the time-resolved MEG representations. However, the peak latencies in Fig 7F demonstrated a hierarchy in the reverse direction over the layers (*R* = −0.79, *P* = 0.05) in comparison with the encoder peak latencies in our proposed model (see Fig 5C). These results replicated the findings by [29] which similarly showed that an untrained AlexNet architecture [39] correlated significantly with MEG representations, but the peak latencies demonstrated a reverse hierarchy compared to an AlexNet trained on object recognition. We further observed that the latent vector representation and decoder layer representations do not show any significant correlations with the brain MEG data (Fig 7E). This shows that the model architecture alone without training can not produce brain like representations in the latent space nor the decoder subnetwork of our proposed model.

When we compared the EVC and IT representations with the untrained model, we found no significant correlations between the encoder layers (E1-E5) and EVC. But, the decoder layers (D1-D4, and D6) showed significant correlations with EVC representations (Fig 8C). None of the encoder/decoder layers showed significant correlations with IT representations (Fig 8D). This shows that the architecture alone can induce representational similarity between the decoder layers and EVC, but not with the encoder layers and not with IT. Therefore, the proper training procedure is crucial for the emergence of brain-model representational similarities.

Together, we found that while architecture alone or non-generative model induces some similarities between the brain temporal and spatial representations, both architecture and training procedure of our proposed model are crucially contributing in emergence of model representations that disentangles feedforward and feedback representations in the visual brain.

## Discussion

Using a generative model, we dissected the dynamics of processes in the ventral visual pathway into two temporally distinct stages: the initial sweep depicts neural representational similarity with the hierarchy of representations along the encoder sub-network which transforms visual features into a latent representation (feedforward sweep); and the subsequent sweep shows neural representational similarity with the decoder sub-network which layer by layer reconstructs visual details from the latent representational code (feedback sweep).

As demonstrated in Fig 5A and 5C, the temporal representational similarities of the encoder sub-network replicates previous findings showing hierarchical representational similarities between the visual brain and the feedforward DNNs that are trained on object recognition tasks [21, 26, 27, 29, 32, 40]. Specifically, it has been demonstrated that object recognition DNNs develop internal representations that are hierarchically similar to brain representations in early visual cortex [40], area V4 (a mid-level region), and IT cortex (a high level categorical region) along ventral visual pathway in primates [21, 26] and humans [27, 29, 32]. Further, comparing the representational dynamics of the decoder sub-network and the visual brain revealed a temporally subsequent hierarchy of processing (Fig 5B and 5C) which progressively builds visual detail from the latent representation. This indicates that beyond feedforward sweep, brain visual processes demonstrate similarities with the reconstruction function implemented in the decoder sub-network. This finding contributes to unraveling the algorithmic functional role of feedback processes in the visual cortex. Representational comparison of two brain regions at the beginning and end of ventral visual pathway, EVC and IT, with the encoder and decoder subnetworks (Fig 4) revealed the encoding processes in the feedforward sweep develops a categorical representations similar to IT and the reconstructing processes in feedback sweep evolves into detail representations similar to EVC. Finally, investigating the impact of model architecture and training procedure, we found that these factors are crucial in the brain-model relationships we observed in this study. specifically, the results suggest that the generative property of our model contributes to the spatial and temporal relations that disentangled feedforward and feedback representations in the visual brain.

## Materials and methods

### Ethics statement

#### Participants

Brain data were acquired from fifteen right-handed healthy participants with normal or corrected to normal vision in two separate experiments (MEG and fMRI). Participants (9 females, 27.87 ± 5.17 years old) signed an informed consent form and were compensated for their participation. Both experiments were conducted in accordance with the Declaration of Helsinki and approved by the Institutional Review Board of Massachusetts Institute of Technology.

Our study consists of two components: (i) The computational model and (ii) the MEG/fMRI data from human participants, both of which are analyzed and compared in the result section. We focus on the ventral visual pathway, hence, we acquire both human brain and computational model neuronal activations on visual centric tasks. In this section, we will describe the computational model, Super Category image dataset, and MEG/fMRI data acquisition and analysis.

### Neuroimaging experiments

The fMRI and MEG data used in this study has been published in [13] previously and is publicly available at http://twinsetfusion.csail.mit.edu/. In this section, we briefly describe the experiment design, data acquisition and analysis.

#### Stimulus set and experimental design

The stimulus set consists of 156 natural images of four distinct visual categories: (i) Faces, (ii) Animates (animals and people), (iii) Objects, (iv) Scenes [13]. The participants viewed images presented for 0.5 sec (with 2.5 sec inter stimulus interval (ISI) in fMRI sessions and 0.7 to 0.8 sec ISI in MEG session) at the center of the screen at 6° visual angle. Functional MRI data were acquired in two sessions (11-15 runs in total) and MEG data were acquired in one session of 25 runs. Images were presented once in each run and in random order. The participants were performing a vigilance task of oddball detection.

#### fMRI data acquisition and analysis

The fMRI experiment was conducted at the Athinoula A. Martinos Imaging Center at MIT, using a 3 T Siemens Trio scanner with 32-channel phased-array head coil. Each imaging session started with acquiring structural images using a standard T1-weighted sequence (176 sagittal slices, FOV = 256 mm2, TR = 2530 ms, TE = 2.34 ms, flip angle = 9°) and then 5–8 runs of 305 volumes of functional data (11–15 runs across the two sessions). Gradient-echo EPI sequence was used for functional data acquisition (TR = 2000 ms, TE = 29 ms, flip angle = 90°, FOV read = 200 mm, FOV phase = 100%, bandwidth 2368 Hz/Px, gap = 20%, resolution = 3.1 mm isotropic, slices = 33, ascending interleaved acquisition). For preprocessing of fMRI data, we used SPM software. The preprocessing of functional data included slice-time correction, realignment and co-registration to the first session T1 structural scan, and normalization to the standard MNI space. For multivariate analysis, we did not smooth the data. We used general linear modeling (GLM) to estimate fMRI responses to the 156 stimuli. The events including stimuli conditions and nulls were modeled with event onsets and impulse response function. Further, the motion and run regressors were included in the GLM. Then we convolved the defined regressors with the hemodynamic response function and estimated the beta-values for each stimulus condition. Then by contrasting each image condition with the explicitly defined null condition, we obtained t-mpas per image condition for each participant. For the current study, we investigated two anatomically defined [36] regions of interest (ROIs) along the ventral visual stream, early visual cortex (EVC) and inferior temporal cortex (IT).

We used multivariate analysis and computed pairwise dissimilarities between 156 image specific fMRI responses using 1-Pearson correlation distances and constructed a 156 x 156 representational dissimilarity matrix (RDM) per participant per ROI (EVC and IT). In detail, we extracted t-value patterns corresponding to each image condition from each region of interest, arranged them into vectors. Then we calculated the pairwise distances of the 156 image specific vector patterns. With this process, we obtained a 156 x156 RDM per ROI for each participant.

#### MEG data acquisition and analysis

The MEG experiment was conducted at the Athinoula A. Martinos Imaging Center at MIT, using a 306-channel Elekta neuromag TRIUX system with sampling rate of 1 kHz. The acquired data were filtered by a 0.03 to 330 Hz band-pass filter. We measured the participants’ head position prior and during the recording with 5 coils attached to their head. We then applied a maxfilter for temporal source space separation and head movements correction [41, 42]. For preprocessing of MEG data, we used Brainstorm software [41]. We extracted trials from -200 ms to 1000 ms with respect to image onset. We then removed the baseline mean and smoothed the data with a 30 HZ low-pass filter. For each participant, we obtained 25 trials per image condition. We employed multivariate pattern analysis to compute the dissimilarity relations between image conditions [10–13, 29, 43, 44]. At each time point t, we arranged MEG sensor measurements of each image condition into pattern vectors of 306 x N dimension, where N denotes the number of trials per condition. We then randomly assigned the trials of each condition into 8 bins and subaveraged the trials within each bin to overcome computational complexity and reduce noise. Support vector machine classifiers were trained on the subaveraged MEG pattern vectors of each pair of images at each time point to discriminate the pairs. The performance of the classifier in discriminating each pair of images with leave-one-out cross validation procedure was used as the dissimilarity measure between the pairs to populate a 156 x 156 representational dissimilarity matrix (RDM) at each time point. The rows and columns of the RDM are indexed by the image conditions and each matrix element indicates the dissimilarity of the corresponding image conditions based on MEG measurements of the specific time point.

### Proposed computational model architecture and training

Previous work revealed that discriminative deep convolutional neural networks trained on object recognition develop similar representations akin to the hierarchical processes along ventral visual stream [3, 26–32]. However, there are abundant number of feedback connections in ventral visual stream and therefore, these feedforward neural network models may not fully represent the complex visual processes in the ventral visual pathway.

Here, we aim to investigate whether a deep generative model trained to map images to a latent code and then reconstruct the images from the features encoded in the latent space can reveal similar representations as feedforward and feedback processes in the ventral visual pathway. With this aim, we developed a deep generative autoencoder model using Adverserial Autoencoder (AAE) framework [34].

AEs consists of two major components: (i) an encoder which takes a given data and outputs a low dimensional representation of the input data (latent code) and (ii) a decoder which takes the latent code and aims to reproduce the input data. Vanilla AEs are often trained with the goal of reducing dimensionality of the the input data by introducing a bottleneck (latent code) within the intermediate layers and minimizing an objective that aims at reconstructing the input data:

$$\begin{array}{c} L=||x-{f\left(x\right)||}^{2} \end{array}$$ (1)

where *f*(*x*) is the output of the model when the input data is *x*.

Although, AEs are generally successful at reconstructing data with high quality, often because of the high degree of freedom over the latent code, the training objective leads to a severe overfitting in the latent space. That is, a small subset of the latent space which is identified by the encoder will yield meaningful content once decoded. However, if a random latent code is fed into the decoder, with high probability it will reproduce a meaningless content.

Therefore, we employed an alternative deep generative autoencoder model called Adverserial Autoencoder (AAE) framework [34]. Original work on AAE [34] utilized only fully-connected layers to perform the task. However, in our proposed model we build on AAE by introducing convolutional layers to better capture the complexity of natural images [39]. Each convolutional block encompasses batch normalization, convolution, nonlinear activation function (Leaky Rectified Linear Unit), and pooling operations (Fig 1). AAE employs an adversarial training procedure to match the aggregated posterior of the latent code with the prior Gaussian distribution. Similar to [34], we incorporated label information in the adversarial regularization to better shape the distribution of the latent code (Fig 1). During the training, for real samples, we provide a one-hot code of its corresponding class label. Alternatively, for the fake samples, we randomly draw a one-hot code from a Gaussian distribution.

To adversarially train our model, we utilize generative adversarial network (GAN) framework. The GAN framework is a min-max adversarial game between two distinct neural networks: (i) The generator (*G*), aims at generating synthetic data by learning the distribution of the real data and (ii) the discriminator (*D*), aims at distinguishing the generator’s fake data from real data. The generator uses a function *G*(*z*) that maps samples *z* from the prior *p*(*z*) (normal distribution) to the data space *p*(*x*). *G*(*z*) is trained to maximally confuse the discriminator into believing that samples it generates come from the data distribution. The solution to this game can be expressed as following [35]:

$$\begin{array}{c} \underset{G}{\text{min}}\;\underset{D}{\text{max}}[\;E_{x∼p_{data}}\left[log\;D\left(x\right)\right]+\;E_{z∼p_{z}}\left[log\;\left(1-D\left(G\left(z\right)\right)\right)\right]] \end{array}$$ (2)

We hypothesize that the encoder embodies similar neuronal characteristics as the image classification DNNs and thereby could resemble the human brain feedforward representations. Alternatively, we hypothesize that the decoder part of the AE architecture which generates the image from the latent space code would encompass the neuronal representations similar to feedback processes in the human visual brain. Our generative autoencoder model architecture consists of a total of 13 layers: (i) the encoder consists of 6 layers; 5 convolution layers and one fully-connected layer, (ii) the decoder consists of 6 layers; 5 transpose convolution layers and one fully-connected layer, and (iii) lastly one fully-connected layer representing the latent code layer. The latent code vector z captures high level representation of the data distribution. The discriminator architecture consists of a total of three fully connected layers (Fig 1B).

### Super category (SC) dataset

In computer vision literature, deep neural network models are usually trained to optimize category specific recognition performance on large scale datasets. However, the human brain learns to develop high-level representations for categories across multiple recognition tasks (eg faces, objects, scenes, etc). Indeed, years of cognitive neuroscience has demonstrated brain regions which functionally respond preferentially to one of these categories compared to others (eg. Fusiform gyrus, IT area, Parahipocampal cortex, …). Therefore, to train our proposed model we put together a super category (SC) data set consisting of 1,980,00 images from four equally distributed distinct categories: (i) Animals, (ii) Objects, (iii) Scenes, and (iv) Faces. Images from the Faces category were acquired from the VGGFaces2 dataset [45], Objects and Animals categories were from the ImageNet dataset [46], and Scenes categories were from the Places356 dataset [47]. To make all classes equal, we have randomly sampled 495,000 images per class. During computational model training and testing, each image was preprocessed through a pipeline: (i) Images were resized to 224 × 224, and (ii) normalized from 0 to 225 to −1 to 1 range values.

Lastly, the neuronal representations for the generative AE model were computed at each convolution/transpose convolution block after feeding 156 images used in the neuroimaging experiments to the encoder of the computational model. Please note this image set was not used in training the model. We employed the vectorized model activity patterns of each convolutional/transpose convolution block to compute dissimilarity distances (1-Pearson’s R) for each pair of images and create an RDM per model layer (Fig 3B).

### Representational similarity analysis to relate the brain and model representations

We used representational similarity analysis (RSA) [37, 38] to map MEG measurements, fMRI responses, and model activation patterns into a common space where they are directly comparable.

RSA transforms the stimulus-specific response patterns into a representational space by creating matrices summarizing pairwise distance relationships of the response patterns (i.e. defined as the correlational distance, or a classifier performance in discriminating two conditions). The matrix capturing these pairwise dissimilarity measures is called representational dissimilarity matrix (RDM).

To relate the spatio-temporal dynamics of neural representations in the human brain with our proposed model, we computed the similarity (in terms of Spearman’s R) of fMRI ROI RDMs and time-resolved MEG RDMs with our computational model RDMs (Fig 3D).

Correlations between subject-specific time-resolved MEG RDMs and computational model layer RDMs result in a signal for each layer per participant across time (Fig 3F). While, correlations between subject-specific fMRI ROI RDMs and computational model layer RDMs result in subject-specific correlation values per layer (Fig 3E). To account for different levels of noise in brain ROIs, we estimated the noise ceiling in EVC and IT [48, 49] and normalized the fMRI ROI and model correlations with the corresponding noise ceiling [50]. Then the correlation time series (for MEG/Model comparisons) or correlation values (for fMRI/Model comparisons) were averaged over participants and tested against zero for statistical significance.

### Statistical tests

We used nonparametric statistical test methods which make no assumptions on the distribution of the data [51, 52]. For statistical inference on the correlation time series, we used permutation-based cluster-size inference with null hypothesis of zero. For statistical assessments of peak latencies, we bootstrapped the subject-specific correlation time series for 1000 times to estimate an empirical distribution over peak latencies [12, 29, 44].
